# Examining the Multi-Scalar Unevenness of High-Quality Healthcare Resources Distribution in China

**DOI:** 10.3390/ijerph16162813

**Published:** 2019-08-07

**Authors:** Meng Yu, Shenjing He, Dunxu Wu, Hengpeng Zhu, Chris Webster

**Affiliations:** 1 Department of Urban Planning and Design, Faculty of Architecture, The University of Hong Kong, Pokfulam, Hong Kong, China; 2 Shenzhen Institute of Research and Innovation, The University of Hong Kong, Shenzhen 518057, China; 3 Public Policy Research Center, Chinese Academy of Social Sciences, Beijing 100732, China

**Keywords:** high-quality healthcare resources, evaluation framework, 3-A hospitals, hospital quality, general entropy indexes, multi-scalar unevenness

## Abstract

Healthcare disparity is, to a large extent, ascribable to the uneven distribution of high-quality healthcare resources, which remains insufficiently examined, largely due to data unavailability. To overcome this barrier, we synthesized multiple sources of data, employed integrated methods and made a comprehensive analysis of government administrative structures and the socio-economic environment to build probably the most inclusive dataset of Chinese 3-A hospitals thus far. Calibrated on a sample of 379 hospitals rated by a reputable organization, we developed a realistic and viable evaluation framework for assessing hospital quality in China. We then calculated performance scores for 1246 3-A hospitals, which were aggregated and further analyzed at multiple scales (cities, provinces, regions, and economic zones) using general entropy indexes. This research shows that the fragmented governance and incoordination of “*kuai*” and “*tiao*” is rooted deeply in China’s legacy of centrally-planned systems, and has had a far-reaching yet partially contradictory influence over the contemporary distribution and performance of healthcare resources. Additionally, the unevenness in the distribution of healthcare resources is related closely to a city’s administrative rank and power. This study thus suggests that the policy design of healthcare systems should be coordinated with external socio-economic transformation in a sustainable manner.

## 1. Introduction

The most recent state-led healthcare reform in China launched since 2009 is characterized by heightened government financial support to tackle the increased disparity in healthcare resource, particularly high-quality healthcare resource distribution. According to the Chinese Ministry of Finance, in 2014 China spent $133 billion on healthcare, although high-quality healthcare services still remain in short supply across the country. There are over 1000 hospitals in China classified as “top-ranked” under a complicated rating system that takes into consideration the academic credentials and experience of the doctors, as well as the equipment and facilities [1]. This number is clearly disproportionate to the 1.4 billion Chinese population. As a result, public hospitals struggle to provide qualified healthcare services to those in need, being overwhelmed by the huge demand. 

In present-day China, the mismatch between the increasing demand for, and the inadequate supply of, affordable and effective healthcare resources has become one of the most pressing social problems, attracting significant attention from the general public [2]. It is well-documented that geographical disparities of healthcare have been (re)produced and consolidated to different degrees and scales [2,3,4]. If the current supply of quality healthcare cannot be improved, the disparity will le worsen, and the government’s commitment to improve the national health scheme outlined in “Healthy China 2030” will become unattainable [5]. As a consequence, the government has put healthcare at the top of the country’s policy-making agenda, and is demonstrating a strong willingness to address the three apparent challenges within the healthcare system, which are, respectively, insufficient healthcare resources (especially those of high quality), the growing disparity in healthcare across different geographic locations, and the rise of cross-region patients seeking satisfactory medical services [6].

One aspect of the uneven geographical distribution of healthcare resources (especially those of high quality) is the agglomeration of large health institutions in the large, more developed cities. This creates geographical accessibility barriers for those who live in rural and less-developed areas. As well as facing longer travel times and higher costs, poorer geographical access can affect the willingness to engage with healthcare, including treatment and follow-up. The idea of a spatially attenuated demand curve for travelled-to goods and services is a well-established phenomenon in all kinds of geography, including health geography. For example, survey-based research reveals that rural residents tend to use hospital services less than urban residents and that rural seniors, minority Chinese, and residents of less-developed regions all make fewer physician visits (mid and Western China) [7]. On the other hand, poor access for the seriously sick has another effect: Migration of patients to the large cities. Regional unevenness in the supply and quality of healthcare resources, as documented in numerous news reports, has caused a large volume of migrant patients to receive medical treatment outside their registered residences.

Attaining a systematic understanding of healthcare disparities is crucial for the government to formulate equitable and sustainable policies on medical insurance, healthcare resources allocation, and disease prevention. To this end, this paper applies co-integration methods to evaluate the performance of top ranked 3-A hospitals and to measure the degree of unevenness in the spatial distribution in China at multiple scales. To overcome the barriers of data unavailability, we synthesize multiple sources of data, employing econometric and entropy index methods and making an integrated analysis of the mechanism of healthcare resources distribution from the supply side, i.e., administrative and institutional considerations. One of the authors has been actively involved in China’s healthcare system’s research for years, serving as a key member of numerous national and local think tanks targeting medical reforms, and his rich experience and well-recognized authority helps validate and reconfirm the methodologies and key findings of this research.

In the remainder of the paper, we first revisit the lasting problem of health disparity and institutions of healthcare resources distribution in different contexts, including China. Section 3 introduces the data, methodology on the rating model, and disparity measurements, while Section 4 presents the results and Section 5 concludes the paper. 

## 2. Revisiting Health Disparity and Institutions of Healthcare Resources Distribution 

### 2.1. Health Disparity and the Uneven Distribution of Healthcare Resources 

Health disparity exists in various dimensions, e.g., differentiations in health utilization among different social groups and divergences in geographical provision of public healthcare service [8]. Institutional barriers, individual characteristics [9,10,11], geographical dispersion, and poor geographical access to health resources [12] can all reduce health equity. To mitigate health disparity, national governments and international organizations have made efforts to reduce gaps between the best-off and worst-off groups in society [13,14]. High-profile efforts include, for example, the World Health Organization’s (WHO) Commission on the Social Determinants of Health, which focused on health inequalities within and between countries [15]; the program to eliminate health inequalities outlined in the Healthy People documents in the U.S. [16,17]; and the UK’s goal to reduce health inequalities in infant mortality and life expectancy [18,19]. In the past two decades, the need for comprehensive interventions to address health disparity has been constantly advocated for by policy makers in diverse settings around the world.

In most affluent countries, general healthcare services are distributed relatively equally. For instance, UK’s National Health Service system is a welfare-guided healthcare at the national level, with allocation principles largely based on a pro-rata equal distribution of this taxation-funded public health service. In some Organization for Economic Co-operation and Development (OECD) member countries, healthcare services also have a pro-poor emphasis, where the access to good-quality physician services is ensured at a relatively low and sometimes zero financial cost at the point of delivery. The distribution of high-quality healthcare services such as specialist care, however, tends to make the total health utilization somewhat pro-rich. This phenomenon appears to be universal and is reinforced when private insurance or private care options are offered, e.g., in the U.S., where a market-driven national healthcare system is dominated by commercial medical insurance and operated on market rules. Market-driven systems tend to enable higher income groups to obtain high-quality and efficient medical services, but generate inequity among citizens with poor health conditions, especially chronic diseases and low income [20]. For low and middle-income countries, including China, the uneven distribution of healthcare resources remains a major challenge [21]. In a vast country like China, given the uneven population distribution and the unequal regional development, various types and degrees of regional disparity in the distribution of healthcare resources are commonly observed [22]. From the established literature, healthcare disparities are well documented among different social groups, e.g., elderly and migrants [11,12,23,24,25,26,27,28], or in the level of healthcare utilization among urban and rural residents [2,3,4]. Using geographical accessibility (service location in relation to users) and related measures of availability [9,26,27,28,29,30,31,32], several frameworks have been proposed based on demand and supply side analyses, and on accessibility considerations to understand the barriers preventing access to healthcare. In regards to reducing institutional barriers, the World Health Organization (WHO) has listed three principles that offer guidance to the distribution of medical resources [23,32]: (a) Distribution according to need (based on Aristotle’s view on distributive justice), (b) distribution by merit or qualification, and (c) the utilitarian principle of distribution (meaning that collective or societal gains have priority over individual gains). In practice, social welfare driven (such as UK, Canada, Sweden) and market driven distribution (e.g., the U.S.) healthcare resources combine in various ways to deliver healthcare in most developed countries. In the previous decades, China has tried to strike a balance between the government and market-provision of healthcare services. It is therefore important to understand the institutional frameworks of healthcare provision and its evolution over time. 

### 2.2. China’s Healthcare Institutions: From Central Planning to Radical Marketization

China’s transitional market economy, with its strong starting point of central planning under an urban–rural dual healthcare system, is creating its own unique blend of institutions. This explains why China’s public hospitals have complex governance structures [33]. Even today, not only is the National Health and Family Planning Commission (NHFPC) responsible for people’s health, but various other ministries also have the power to allocate sectorial funds for healthcare, to set prices and payment methods, and to decide on human resource allocation and capital investment. Competing ministries often pursue their own bureaucratic interests, and issue policies and regulations that contradict the socioeconomic goals of public hospitals. In short, the Ministry of Health has the responsibility for healthcare provision, but by no means monopolizes its provision [33]. In this connection, the conflicting governance structure of China’s healthcare resource is a key issue. Notably, the evolution of China’s healthcare distribution system has also been significantly reshaped by the country’s economic reforms. To clearly understand China’s healthcare institutions, we first review the evolving healthcare system by examining the stakeholders in the supply of healthcare resources in the following section. Based on this, we identify key indicators for the modeling of healthcare performance, which are used later in this study.

In the pre-reform period (1949–1978), a combination of the “*danwei* (work unit)-based” healthcare security system in the urban areas, consisting of Government Insurance Scheme (GIS) and the Labor Insurance Scheme (LIS), and the “collective-based” system in rural areas, i.e., the Cooperative Medical Scheme (CMS), covered the entire nation. Given the lack of a market economy in the pre-reform era, public institutions were the main actors in the supply and allocation of healthcare services. This supply system was vertically organized within the administrative hierarchy, known as a “*tiao*”, meaning the vertical/sectorial lines of authority over various sectors reaching down from the ministries of the central government to the provincial and prefectural organizations. Each “*tiao*” was able to establish its own healthcare system for the purposes of self-sufficiency, so that public hospitals within these bureaucracies were established, including railways hospitals, mining and petroleum hospitals, military hospitals, etc. Meanwhile, the horizontal/territorial level of authority, known as “*kuai*”, had different levels of functional departments that organized, operated, and managed medical institutions and resources within their jurisdiction, e.g., directly affiliated provincial or sub-provincial leveled hospitals. Given that, in those years, China had been struggling with poverty alleviation and the priority of healthcare was disease prevention (especially epidemics and infectious diseases in rural areas) rather than equitable and effective treatments, healthcare institutions were largely planned based on population size and the responsibilities of healthcare services provision were devolved to sectorial and territorial units. In other words, people’s access to healthcare was decided by their sectorial affiliations either within the system (*tizhinei* in Chinese), i.e., government-affiliated or state owned enterprises, or outside of the system (*tizhiwai* in Chinese), i.e., private sector or self-employed, or unemployed and territorially bonded residential registration (urban or rural hukou). The rigid and un-coordinated “*tiao*-*kuai*” matrix of healthcare provision unsurprisingly resulted in multi-authority management, redundancy in hospital construction, and inefficiency in the supply and utilization of healthcare resources [34]. 

In the post-reform period (after 1978), the Chinese government gradually transferred local service provision powers from central governments and state owned enterprises to local governments to expand locality autonomy in policy-making at the same time as cutting down the financial budget allocation as a way of encouraging marketization. This deeply affected the public healthcare supply system. Changes started to occur in the medical insurance system in 1985: The LIS and GIS programs were merged by the central government under the reformation of the Chinese state owned system, and the Medical Savings Accounts scheme (MSA) was created as a new urban employee health insurance program. In rural areas, the original CMS collapsed and payments came to rely on households’ out-off-pocket resources. A wave of nation-wide medical reforms carried China’s hospitals into a new episode of market-oriented and profit-driven transformations, after “The Opinions on Deepening the Reform of the Public Healthcare System” was issued by the State Council in 1992, which aimed at a partial transfer of the functions of traditional public hospitals to the market. This medical reform generated new economic and political incentives that guided the behavior of officials in the “*tiao*-*kuai*” matrix [35]. This resulted in fierce competition for patients among hospitals in the matrix of “*tiao*” and “*kuai*” systems, and triggered a series of adverse outcomes, such as over-prescription and price discrimination against certain patients, heavy investment in advanced medical equipment, and inefficient expansion of hospital capacity and scale. As Yip and Hsiao (2009) observed, this led to fragmented governance, distorted human resource deployment, overuse of drugs and diagnosis for revenue generation, as well as regional and socio-economic inequalities.

In 2003, the SARS epidemic exposed the weaknesses of its multi-channel administration and unresponsive healthcare system [36]. China has gone through a period of policy reflection from 2003 to 2008, in which the gains and losses of the healthcare transformation came under close scrutiny and probing discussion led to a new round of healthcare reforms in 2009. New reforms were based on three fundamental tenets: A strong role for the government in the provision of healthcare; a commitment to equity in healthcare provision and medical insurance; and a willingness to experiment with regulated market approaches in terms of drug purchases and price control [21]. In this regard, four principal policies were designed to resolve the uneven geographical distribution of high-quality healthcare resources: The Tiered Health Care Delivery System (THCDS); Direct Transaction of Health Security for Cross-region Patients (DT); Medical Partnerships (MP); and National and Regional Medical Centers (NRMC). Their outcomes have not yet been systematically evaluated at the national level.

## 3. Data and Methodology 

Though many researchers have stated that the uneven distribution of healthcare resources among different social groups is significant in China after 2009 [11,12], most focus only on a single or a few dimensions of these resources (e.g., healthcare institutions, personnel, facilities, etc.). Furthermore, their sources of data are mainly census data or yearbooks, which are usually at an aggregated scale and provide no performance information on hospitals [37,38,39]. Given the constraints in data accessibility, presenting a full picture of the distribution of high-quality hospitals in China requires innovative feasible methodologies in data collection and modeling. In our study, we have sought to explore the disparity in healthcare resources using integrated datasets and a more comprehensive and feasible methodology than other studies, in order to evaluate hospitals’ performance and the distribution of high-quality healthcare resources.

### 3.1. Developing an Integrated Database of High-Quality Hospitals 

**Figure 1:**
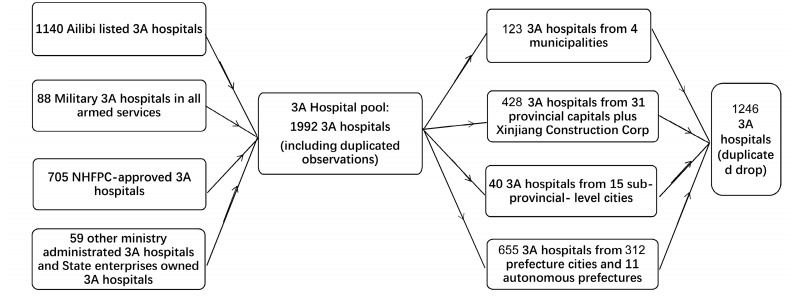
Data Collection and the Data Sorting Process. Source: Compiled by the authors.

We began by creating integrated data on high-quality hospitals. In China, the officially recognized standard of “high-quality” medical resources is China’s Tertiary and Ten-Classes Hospital System. According to the “Measure for the Administration of Hospital Grades” issued by the NHFPC in 1989 (the Measure hereafter), hospitals are graded from high to low, respectively, as tertiary, secondary, and primary, and each can then be further divided into three classes (from high to low: Class A, B, and C). According to the *Measure*, hospitals at a Tertiary Grade of A or A^+^ Class (the 3-A hospitals hereafter) can be taken to be high-quality hospitals. Due to the institutional fragmentation associated with the “*tiao*-*kuai*” systems introduced above, 705 (including non-public) out of a total of 5472 registered hospitals were officially identified as 3-A hospitals by the NHFPC, according to the NHFPC database published in December 2017. However, as the NHFPC database excludes hospitals affiliated to other “*tiao*” systems, this database only partly accounts for the high-quality healthcare resources in the country. Our study seeks to include as many high-quality hospitals as possible, and therefore we collected 3-A hospitals from other “*tiao*” systems, e.g., military hospitals, which are administrated by the Health Sector under the General Logistics Department of the People’s Liberation Army of China (PLA); armed-police and fire-fighting hospitals, which are affiliated to the Ministry of Public Security; hospitals affiliated directly to the Xinjiang Production and Construction Corps (XPCC), which is considered as a separate province in this research, for its unique economic and paramilitary organization with a sub-provincial status that independently fulfills governmental functions (e.g., healthcare and education); and hospitals affiliated to major Ministries, which still play an irreplaceable role in the national healthcare service system. Establishment, ownership, and other indicators for all the above-mentioned 3-A hospitals have been collected to form a comprehensive dataset containing a total of 1246 3-A hospitals. Even though we have tried our best to develop a complete dataset, it is inevitable that the observations on hospitals might have some selection bias due to accessibility and time constraints, particularly incomplete coverage of *tiao* system hospitals. Figure 1 shows how the information and data were collected, classified, and aggregated. 

Assessments of the quality of China’s 3-A hospitals are rare in the literature. Our evaluation framework for measuring the quality of 3-A hospitals mainly makes reference to several reputable and available hospital ranking systems that grade hospitals based on an assessment of their facilities, establishments, specialties, etc. Among them, we compared the four most reputable hospital ranking lists, i.e., the Best Hospitals in China, published by Fudan University; the Blue Book of Hospitals-Annual Report on China’s Hospital Competitiveness, published by Ailibi Inc. (a reputable hospital management and consulting institute, the Ailibi-2016-ranking hereafter); the Best General Public Hospital in China, published by Sichuan University; and the Top 100 High-tech Hospitals in China, published by the Chinese Academy of Medical Sciences and Peking Union Medical College (CAMS-PUMC). We chose the Ailibi-2016-ranking as the key reference for developing our own evaluation framework. This choice was based on the fact that, of the four lists, it covers the largest number of hospitals (2199 hospitals of different levels and classes, under six categories). In addition, Ailibi-2016-ranking is highly correlated with both the Fudan Ranking and the CAMS-PUMC Ranking according to a two-sample Kolmogorov-Smirnov test, that we conducted. However, Ailibi-2016-ranking on its own was not completely suitable for our study for a number of reasons. First, only the scores for the top 100 out of 500 hospitals under each category are reported. Second, the information and criteria used by Ailibi-2016-ranking to build its evaluation model is not available. Third, the Ailibi-2016-ranking grading system places significant emphasis on operational input and output, while giving less consideration to other hospital attributes important for China’s healthcare institutional/administrative system. We therefore extracted 379 3-A hospitals with specific scores from Ailibi-2016-ranking to be further analyzed to develop our own evaluation framework.

### 3.2. Modeling a Comprehensive Evaluation Framework

Using the 379 scores from Ailibi-2016-ranking as the dependent variable, we included key factors affecting hospital quality identified in Section 2.2 above as independent variables to model the mechanism of the “*tiao*-*kuai*” system from three dimensions, with other major variables taken into account.

The first set of independent variables includes basic information on 3-A hospitals, e.g., facilities, establishment, and the population and economic status of the city in which the 3-A hospital is located. Using the STATA Stepwise regression method, three key variables were identified out of a total eight: Total number of hospital staff (*var*1), permanent population of the city (*var*2), and the city’s average disposable income (*var*3). We also included other important attributes: Ownership (*var*4), medical treatment style (*var*5), and historical factors, i.e., church and charity-initiated hospitals (*var*6), which were the earliest Western hospitals and the origins of Western medicine in China, and evolved into many major 3-A hospitals nowadays.

A second set of variables was developed based on the vertical administration systems of *“tiao”* with a focus on three major sectors: Medical universities/research institutions affiliation (*var*7), Health, and Family Planning Commissions (HFPCs) at different administrative levels (*var*8, *var*9, *var*10, and *var*11) and military hospitals (*var*12). 

A third set of variables included the jurisdiction system of “*kuai*”. We use the city’s administrative level (*var*13) as a marker of the city’s position among the four levels, with the order of the four levels, respectively: Centrally-administered municipality, provincial/autonomous region capital, sub-provincial level city, and prefecture city/autonomous prefecture. For 3-A hospitals located in counties, we aggregate them to their directly affiliated prefecture cities.

The estimated best-fit model based on available data is as follows:

(1) $$\begin{array}{l} 3A\;Hospital\;Score_{i}\\ \;=\beta_{0}+\beta_{1}Hospital\;Staff_{i}+\beta_{2}City\;Permanent\;Population_{j}\\ \;+\beta_{3}City\;Disposable\;Income_{j}+\beta_{4}Hospital\;Owership_{i}\\ \;+\beta_{5}Medical\;Treatment\;Style_{i}+\beta_{6}Church\;\&\;Charity\;Initiated_{i}\\ \;+\beta_{7}Military\;Hospital_{i}+\beta_{8}NHFPC\;at\;Different\;Levels_{i}\\ \;+\beta_{9}(University\;Affiliation_{i}\;\times \;City\;Administrative\;Level_{j})\\ \;+\beta_{10}(NHFPC\;at\;Different\;Levels_{i}\times \;University\;Affiliation_{i})\\ \;+\epsilon_{i} \end{array}$$

where $i=1,\ldots ,M\left(M=379\;\mathrm{hospitals}\right),j=1,\ldots ,N\left(N=312\;\mathrm{cities}\right),\;\mathrm{and}\;\epsilon_{i}$ is the error term.

Detailed information and data sources of all variables are reported in Table 1. The statistics of the estimated model (Equation (1)) are reported in Table 2. The adjusted R-square of the model was 59.59, with a *VIF* of 6.56, which suggests that multi-collinearity is negligible. Major findings are reported in Section 4.

### 3.3. Measuring Uneven Distribution of High-Quality Healthcare Resources 

In employing this evaluation frame, we used the data collected for the remaining 867 3-A hospitals to predict their scores and obtained a total of 1246 scores (the full score list of the 1246 3-A hospitals is available on request). Referring to the China City Statistics Yearbook in 2016, we aggregate the scores at city level for 312 cities at all four administrative levels. This is because, besides the 297 cities listed by China City Statistics Yearbook of 2016, we added an additional 14 autonomous prefectures and XPCC to the dataset to create a more inclusive list, thus yielding a total of 312 cities. Using general entropy inequality measurements, and decomposing them by sub-groups, we further analyzed the variations at multiple scales, as detailed in Section 4.

#### 3.3.1. Inequality Measurement Methods

The Gini index and generalized entropy (GE) measurements [40,41,42] such as the Theil approach are most commonly applied to measure levels of inequality/unevenness. The Gini index denotes the overall inequality that exists at a certain point in an orderly accumulated percentage of the population [43], but since it is a systemic measure, it cannot be decomposed if one is interested in the level of inequality among different sub-populations. For the sake of decomposability and statistical testability, we adopted the GE measurement method. Each GE (α) index can be decomposed as $I_{total}$ = $I_{within}$ + $I_{between}$, where $I_{within}$ is the within-group Inequality, as the weighted sum of the inequalities within each subgroup; and $I_{between}$ is the between-group Inequality, as the proportion of a city’s score derived from the mean score of the subgroup to which the city belongs [44]. We used within-group inequality to examine the disparity occurring within each subgroup, based on hospital scores; and between-group inequality was calculated to examine differences between different sub-groups.

The GE family of measures is given by Reference [45]:

(2) $$GE_{\left(\alpha \right)}=\frac{1}{\alpha \left(\alpha -1\right)}\left[\frac{1}{N}\overset{N}{\underset{i=1}{\sum }}{\left(\frac{y_{i}}{\bar{y}}\right)}^{\alpha }-1\right]$$

where $\bar{y}$ is the mean score of all 3-A hospitals in a city per 10,000 people; $y_{i}$ is the score of 3-A hospitals for the $i^{th}$ 10,000 people; $\frac{1}{N}$ is the population share $y_{i}$ takes in the total population; and *N* is the total population. Parameter α represents the weight given to the distances between scores located at different positions of the score distribution, which can take any real value. For lower values of α, GE is more sensitive to changes in the lower tail of the distribution, and vice versa. As *α* increases, the influence of the top scores upon the index increases, as does the influence of the lower scores as α decreases [46]. There are two special cases of a more flexible general class that are based on appropriate normalization: When α = 0, the index is not sensitive to changes in the distribution of scores, known as Theil’s L, or the mean logarithmic deviation (MLD), see Equation (3):

(3) $$GE_{\left(0\right)}{\mathrm{Theil}}^{’}s\;L=\frac{1}{N}\overset{N}{\underset{i=1}{\sum }}\ln\left(\frac{\bar{y}}{y_{i}}\right)$$

When α = 1, the index is known as Theil’s T index (Equation (4)):

(4) $$GE_{\left(1\right)}{\mathrm{Theil}}^{’}s\;T=\overset{N}{\underset{i=1}{\sum }}\frac{y_{i}}{Y}\ln\left(\frac{y_{i}N}{Y}\right)$$

We computed and report both GE (0) and GE (1) in Section 4. 

#### 3.3.2. Inequality Decomposition by Subgroups

We first used provinces and prefectures as the official partition standard. In the literature, the “seven natural geographical regions” (known also as the seven economic corporative regions, include NE-Northeast, NC-North China, EC-East China, CC-Central China, SC-South China, SW-Southwest, and NW-Northwest) and the “three economic development zones” (which include Eastern China, Central China, and Western China) are often used in examining regional disparity [32]. Our decomposition of inequality indices follows these partitions and added to the city-level partition. The decomposition of Theil’s L is shown in Equation (5):

(5) Theil′s L=L0= 1N∑i=1Nln(y¯yi)      =Lwithin+ Lbetween      =∑k(Nk/N)Lk+∑k(NkN)ln(YkNkYN)

For *k* groups that are mutually exclusive and exhaustive, let *Y* be the total score of all *N* populations in the dataset, so y¯=YN will be the population mean score and y¯k=YkNk the mean score of subgroup *k*. Let yk=y¯ky¯ represent group k’s mean score relative to the population mean score, in which $L_{k}$ is the inequality value of Theil’s L for subgroup *k* (which is calculated considering subgroup *k* as a separate population).

The decomposition of Theil’s T is given by Equation (6):

(6) Theil′s T=T1=∑i=1NyiYln(yiNY)       =Twithin+ Tbetween       =∑k(YkY)Tk+∑(YkY)ln(YkYNkN)

where $T_{k}$ is the Theil’s T value for subgroup k. Let YkY be the score share of subgroup *k*, as the share of total scores held by *k*’s members. Given that NkN is the population share of a subgroup *k*, (YkY) /(NkN) represents each population’s share received from the share of the mean of *k* within a given subgroup *k*.

Following Equations (3) and (4), we first computed the city-based inequality value for 308 cities (excluding the four centrally administrated cities) and then decomposed them into three administrative levels using Equations (5) and (6). We further decomposed all 312 cities by 32 provinces (XPCC is treated as an independent province), as explained earlier, and Hong Kong and Macau and seven geographical regions and three economic zones are not included. These analyses were processed using the ineqdeco. ado program in STATA, an inequality indices code with optional decomposition by subgroups using new Stata commands (version15.1, StataCorp, College Station, TX, USA).

## 4. Empirical Results and Major Findings

In this section, we first report the major findings on how the administrative system, specifically the *tiao* and *kuai* system, affect the performance of 3-A hospitals during the process of developing our evaluation framework. We then report the overall distribution of 3-A hospitals and the unevenness of their distribution among different sub-populations based on the inequality indices specified in Section 3.3.

### 4.1. Determinants of 3-A Hospitals’ Performance 

Through modeling the determinants of available hospital scores, we have not only developed a credible innovative evaluation framework for China’s 3-A hospitals, but also identified key factors influencing hospitals’ performance in relation to China’s unique institutional settings and legacies. As shown in Table 2, all of the basic factors of the hospitals (var1, var2, and var3) influence the overall score of 3-A hospitals. This can be attributed to the legacy of China’s centrally planned economy, during which a hospital’s planning and establishment (var1) was mainly in line with the population density in major cities (var2). The positively significant linear relationship that exists between city’s disposable income (var3) and *3-A hospital* score reveals the escalating medical costs over the past three decades.

Furthermore, churches and charitable origins (var6), military hospital (var12), public ownership (var4), and Western medicine style (var5) are all positively and significantly related to the overall quality of 3-A hospitals. Sixty-two out of 379 3-A hospitals originated from a church or other philanthropic organization, for example, Beijing Union Medical College Hospital. As for public Traditional Chinese Medicine (TCM) and hybrid Western medicine/TCM approaches, the negative yet significant coefficients indicate that TCM only plays a limited role in the quality of present-day China’s healthcare system. In fact, TCM had a vital role in the early years when the People’s Republic of China was founded, its practitioners using locally available, less expensive approaches to treatment, especially in rural areas, contributing greatly to the prevention of epidemics and infectious diseases. While China actively promotes the integration of TCM and Western medicine in order to optimize therapeutic effectiveness at a lower cost, and has established a system of TCM education and practice [47], the success of treatment is very much contingent upon the particular disease, population characteristics, and the experience of the physician.

The last finding highlights issues related to “*tiao-kuai*” systems and their interaction under different external environment. Hospitals directly affiliated to universities/colleges (*tiao/*var7) located in four levels of cities (*kuai*/var13) all have a significant and positive impact on the hospital scores. This implies that they have benefited from the affiliation system linking medical universities/colleges with locally affiliated hospitals by way of sharing human resource, technology, professional training, etc. For hospitals affiliated with universities/colleges (*tiao/*var7), but not directly administered by the NHFPC (*tiao/*var8) or prefecture-level HFPCs (*tiao/*var11), hospital scores turn out to be negative and weakened. Given that those affiliated with universities/colleges and directly administered by NHFPC (20 observations) or prefecture-level HFPCs (23 observations) are used as the reference group in the regression, this result suggests mutually independent effects from different “sectors”, i.e., universities and public health authorities under the “*tiao*” system. Those simply directly administered by NHFPC do not show statistical significance, yet the positive sign indicates the importance and value of direct administration by the NHFPC—greater support in personnel, fiscal, and land allocation. This is particularly true for 3-A hospitals directly administered by prefecture-level HFPCs (*tiao/*var11). For those HFPCs at the level of provincial capitals (*tiao/*var9) and municipalities (*tiao/*var10), however, we found a positive but not significant association. These results reconfirm the independent effects at different scales within the “*tiao*” system, partially affected by the “*kuai*” system (from central to local). Resulting from the decentralization process, the public finance systems at different administrative levels are independent of each other. Municipalities and provincial capitals need to finance the public health sector within their jurisdictions, but public health authorities (e.g., HFPCs) in these cities still support their directly administrated 3-A hospitals with discretionary power, e.g., providing land and fiscal subsidies. Overall, the performance/quality of 3-A hospitals is attributed to a series of complex mechanisms under the confluence of the “*tiao*” and “*kuai*” systems.

In summary, our model reveals that, in general, a city’s position in the four-level administrative hierarchy is a fundamental principle in the de facto allocation of 3-A hospitals, as a long-lasting legacy of the centrally planning economy. The quality of hospitals largely reflects the interests and power structure of the vertically organized administrative bodies.

### 4.2. 3-A Hospitals Scores Distribution and GE(α) Values at Multiple Scales

Only 272 out of 312 cities had valid scores, while the other 40 cities (13.5%) scored zero due to the absence of 3-A hospitals. Table 3 lists the top 10 cities based on their aggregated 3-A hospital scores and the average score per 10,000 people, respectively. Notably, the top 10 cities based on their aggregated 3-A hospital scores are the most important municipalities and provincial capitals, with six out of 10 being in the Eastern coastal areas. Nonetheless, among the top 10 cities based on the average score per 10,000 people, cities with a high concentration of 3-A hospitals and a relatively small population size stand out, e.g., Xining, Lhasa, and Urumqi. Cities like Beijing, Nanjing, Shenyang, and Guangzhou appeared in both top 10 lists, showing an absolute advantage in high-quality healthcare resources. 

Table 4 (at city level) and Table 5 (above city level) shows how the GE(α) values based on a city’s average score per 10,000 people varies across different geographical scales. As we “scale up” spatially, i.e., from individual cities to provinces, and to regions and zones, a general trend of unevenness, dominated by within-group disparities, can be identified in the distribution of 3-A hospitals in present-day China. This largely confirms other empirical research, which suggest that, typically, at least three-quarters of the inequality in a country is ascribable to within-group inequality, while the remaining one-quarter is attributable to between-group differences [48].

### 4.3. Interpreting the Unevenness at Different Scales

**Figure 2:**
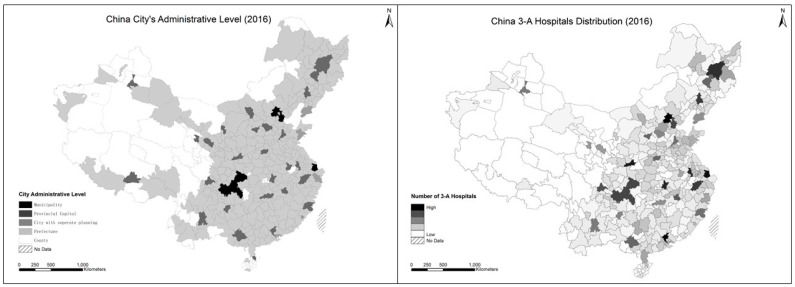
China City’s Administrative Level vs. 3-A Hospitals Distribution. Source: Compiled by authors.

To further interpret the spatial disparities in averaged city scores on multi-layered geographical scales, we mapped the outcomes of GE(0) and GE(1) at multiple scales using GIS tools to visualize the distributions (see Figure 2). As shown in Figure 2, the disparity in cities’ aggregated 3-A hospitals’ index clearly reflects the cities’ hierarchical positions, which implies that administrative hierarchy plays a decisive role in healthcare resource allocation [49]. 

**Figure 3:**
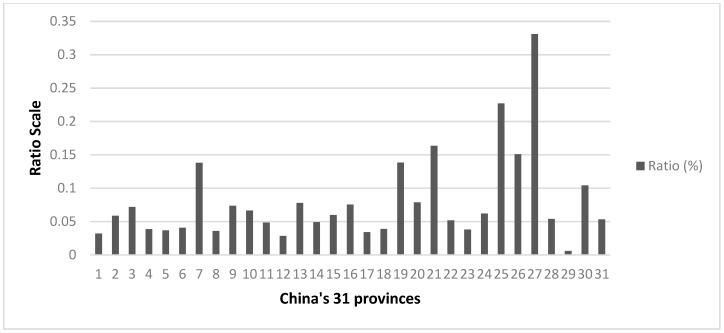
The Ratio of the Number of Patients served by Community-based Healthcare Centers to Patients Served by Tertiary Hospitals across 31 Provinces. Source: China Health Statistics Yearbook (2016); Note: The sequence of the 31 provinces is the same as the province list in Table 5; Xinjiang Production and Construction Corps (XPCC) is excluded here since its data is not recorded as a province in the yearbook.

As we scale-up to a provincial level, by GE(0) or GE(1) as both are highly consistent, we find within-group disparity accounts for about 76%–80% (rising from about 50% at city level) of total inequality. The huge within-group disparity points to the major problem faced by vulnerable provinces where high-quality healthcare services are highly unevenly distributed. The top five most uneven distributions of average hospital scores are found in Jiangxi, followed by Shanxi, Xinjiang, Shaanxi, and Guizhou, all of which are inland or Western provinces with a low level of economic and urban development, as indicated by the lack of “sub-provincial-level cities” or “city clusters” other than the provincial capitals. It is also worth noting that far fewer medical-university/college affiliated 3-A hospitals, a significant factor in the performance of 3-A hospitals [50], are found in these provinces in comparison to coastal provinces. Furthermore, through calculating the ratio of the number of visits at community-based healthcare centers to those at tertiary hospitals for each province in 2016 (see Figure 3), which is an indicator of the allocation and utilization of the healthcare resources between primary and tertiary hospitals, we found a similar pattern of unevenness among these provinces. This “inverted pyramid” structure [50] implies that the community-based healthcare service system in these provinces does not function very well in preventing patients crowding into 3-A hospitals. This may be on account of various reasons leading to inefficient performance in primary healthcare systems in more backward provinces, e.g., lack of experienced and skilled doctors, medical equipment, leaving medical resources unused, and so on. As a result, people have to spend more money and time visiting 3-A hospitals for quality healthcare services. Additionally, for inland provinces, usually with a low level of urbanization, especially provinces with large rural and mountainous areas inhabited by minorities, e.g., Xinjiang and Yunnan, the problem caused by the lack of qualified primary healthcare resources is more prominent, where illness can easily lead to poverty for farming households. Lastly, among eight provinces/municipalities with the most even distributions, where GE(0) = GE(1) = 0, the level of evenness of four of the provinces, Tibet, Ningxia, Qinghai, and XPCC, is likely to be due to lack of rivalry from other prefectures within the province. This implies the absolute concentration of 3-A hospitals in a single city, usually the provincial capital. For instance, in the Qinghai autonomous region, all 3-A hospitals are concentrated in the provincial capital Xining. The same holds for XPCC, which comprises 14 middle-sized cities spread along the border of Northwest China, with all 3-A hospitals agglomerated in Urumqi, where XPCC’s administrative institution is located. For the other four municipalities, their evenness is an apparent result of a “zero” sub-group in the jurisdiction, and thus no variance can be captured.

At a regional level, the division of seven geographic regions is based largely on regional economic development collaborations and geographical proximity and the within-group disparity increases rapidly to about 92% (from 80% at the provincial level). We note that a relatively small share of between-group inequality does not mean that the average difference between them is less important than inequalities within these groups [51]. In particular, social stability and harmony can break down once the average difference between groups goes beyond a certain threshold that varies from country to country [51]. In our research, by GE(0) or GE(1) (both being highly consistent), the most uneven distribution is found in the Northwest region (7-NW), followed by the North region (2-NC) and Central region (4-CC). This finding indicates a high level of disparity prevalent across the six adjacent NW provinces, which is expressed as the high concentration of 3-A hospitals in the provincial capitals, with much fewer found in other prefectures. A closer examination of public financial expenditure data in healthcare shows that this indicator is one of the reasons leading to disparity. In 2016, the average expenditures per capita in Xinjiang (RMB 6294 yuan) and *Tibet* (RMB 7701 yuan) are almost as high as Shanghai (RMB 6444 yuan) and Beijing (RMB 8490 yuan). However, Xinjiang is the province with the third most uneven distribution of high-quality healthcare resource, which further reveals the urgent problems of resource misallocation between urban and rural, primary care, and tertiary hospitals in Northwest China. In the CC region, 3-A hospitals are also concentrated predominantly in the provincial capitals. While slightly different from NW, the rest of the prefectures in CC share a similarly low average score due to their large rural areas and populations. It is noteworthy that the brain drain of human resources seen from primary and secondary healthcare institutions to the Southeast coastal areas (e.g., Guangdong) where there have been greater opportunities for career development over the past two-and-half decades, while the remaining health institutions have not been well developed [52,53]. Without sub-provincial-level cities or city clusters, the overall development of 3-A hospitals in CC has been largely constrained, as human resources have agglomerated strongly in provincial capitals. Turning to NC, the disparity can be largely ascribed to the within-group contrast between the two centrally administrated cities of Beijing and Tianjin, and the inland region, including Shanxi province and the Inner Mongolia autonomous region. Taking Beijing as an example, there are 53 3-A hospitals in the city, according to our available data, which is 1.5 times the total of Shanxi and almost three times that of Inner Mongolia. However, we note that this does not necessarily suggest that Beijing has achieved an optimal healthcare system. Given that the aging population is growing faster in Beijing, than elsewhere, exceeding 24% of the city’s total population by the end of 2016, the ratio of community-based primary care to tertiary hospitals arguably remains too low.

The distribution at the zone level confirms and explicitly shows the trend of high concentration of 3-A hospitals in China’s coastal areas. Western China (3-WC) has the highest level of unevenness, measured by both GE(0) and GE(1), followed by Central China (2-CC) and Eastern China (1-EC). It can further be noted that a relatively small difference exists between Western China and Central China, while the difference between Eastern China and the other two zones is much larger. This can be attributed to the gap in economic development between the coastal areas and inland China, and the finding suggests that Western and central regions deserve a larger share of central government subsidies to finance high-quality healthcare services.

## 5. Conclusions

To deliver a transition in the healthcare system from planned economy to marketization, since the 1980s, a series of healthcare reforms have been enacted. Most notably, the transformative reforms of 2009, underwritten by a strong government role, aimed at tackling the problem of the uneven distribution of high-quality healthcare resources [54]. The contributions of our study, which can be seen as part of this reform endeavor, are two-fold: (1) We first develop a novel method for the evaluation of hospital quality at a national and sub-national scale; and (2) we use it to provide an updated comprehensive understanding of the spatial unevenness of the distribution of 3-A hospitals at multiple geographical scales.

On the eve of “Healthy China 2030”, public healthcare services in China, including both primary care services and general hospitals, although suffering from some deeply rooted problems, still delivered more than 90% of the country’s inpatient and outpatient services, which dropped slightly to more than 80% in 2017, and performing an important function in general outpatient care and earning most patients’ trust [21]. However, in the absence of an authoritative and comprehensive system for the evaluation of hospital performance, 3-A hospitals have been rated mainly based on their “hardware”, e.g., number of beds and number and credentials of their physicians. This may be misleading for both decision makers and patients. The former will tend to increase their support to the high-ranked hospitals, while the latter will tend to continue to flock to the high-ranked 3-A hospitals. This highlights the importance of an effective evaluation framework, without which the existing problem of healthcare disparity may not be adequately exposed and addressed. Accordingly, our research developed a realistic and balanced evaluation framework for the performance of 3-A hospitals. Building an inclusive dataset of Chinese 3-A hospitals from multiple sources, we first developed a comprehensive evaluation framework for the assessment of quality of 3-A hospitals, introducing geographical, historical, and institutional factors such as the “*tiao-kuai*” systems, to complement more conventional evaluation parameters. A regression-based quality model was fitted on a sample of 379 hospitals, and this was then used to estimate the quality of 3-A hospitals nationally, enabling the distributive pattern to be examined at multiple geographical scales.

Two key research findings concerning the uneven distribution of high-quality healthcare resources can be highlighted. First, the fragmented governance resulting from the uncoordination of “*kuai*” and “*tiao*” is deeply rooted in China’s legacy from the centrally-planned system, and has had far-reaching and partially-contradictive influence over the contemporary distribution and performance of healthcare resources [55]. This is manifested in the positive impact on hospital performance resulting from complementary administrations in certain “*tiao*” and “*kuai*” hospitals, e.g., affiliation with medical universities/colleges and different levels of cities. On the other hand, the quality of healthcare can be impaired by the uncoordinated “*tiao-kuai*” administration. It is noteworthy that, in principle, the “*tiao-kuai*” systems can work in a coordinated way when their institutions are designed in a complementary rather than competitive manner and are in line with the external socioeconomic circumstances and actual healthcare demands. Nonetheless, in reality, the uncoordinated “*tiao*” and *“kuai*” administration involved in 3-A hospital management has led inevitably to the inefficient allocation and utilization of scarce resources.

Secondly, the unevenness in the distribution of healthcare resources is related closely to a city’s administrative rank and power: The higher the level, the better and the higher the concentration of quality healthcare resources. This is reflected in the dominant within-group disparity in the overall inequality across different geographical scales. We have shown that unevenness becomes stronger as the geographical scale moves up to the provincial level, and further to the regional level and zone level. Additionally, the imbalance is manifested in different ways in different geographical spaces. For instance, in economically lagging inland provinces, e.g., Xinjiang, a high average financial expenditure in healthcare per capita can co-exist with a very uneven distribution result. 

With China’s demographic structure and socioeconomic environment continuing to evolve along with the deepening of market reforms, governments at all levels are under pressure to adjust their strategies to improve public healthcare systems. For instance, epidemics and infectious diseases have been substantially reduced, yet chronic and non-infectious diseases are on the rise. Meanwhile, citizens’ demands for healthcare services are increasingly diversified according to affordability, health conditions, and perceptions. An allocation mechanism weighted to administrative hierarchy and power is not suitable under these conditions. Simply injecting investment in high-ranked hospitals, while ignoring effective and reasonable provision and use of primary healthcare services will compromise the longer term sustainability of cities as places to live and as economic engines [56]. Finally, the advancement in medical and healthcare technologies, the increasing mobility of doctors, and the emergence of electronic health records (EHR) will bring further challenges to China’s rigid healthcare system, with its roots in the centrally planned economy. If adopted wisely, they may eventually transform the patterns of healthcare resource distribution. It is important to monitor the changing patterns of unevenness across space, time, and social group. Our methodology creates metrics that can do this with comparisons of static and changing inequalities across and between the key spatial scales of healthcare service supply, administration, and governance, and across and between groups of target population demand. That said, we acknowledge the limitations of our study, including the limitation of data availability in respect of, for example, hospital governance structure and quality, revenues and government subsidies. Our models can be improved with a larger sample size and the inclusion of other key variables once relevant data becomes more readily available. Additionally, in the future, incorporating ranked weights and entropy [57] could be another way to further improve the evaluation frame. 

## Figures and Tables

**Table 1:** Variable Summary and Data Source.

Variable Details in Equation (1)	Category	Data Source
y: 379 Ailibi 3A hospital score		Ailibi-2016-ranking
*var*1: No. of total staff (person)		Hospitals’ official websites. For missing information and data, we visit other websites such as www.haodf.com and https://www.jobmd.cn/
*var*2: city2016 permanent population (unit: 10,000 person)		China City Statistical Yearbook 2017
*var*3: city 2016 disposable income (yuan)		
*var*4: ownership	1. public owned 0. non-public owned	Hospitals’ official websites
*var*5: medical treatment style	Western TCM Western + TCM	Hospitals’ official websites
*var*6: church & charity initiated	1. with church or charity as predecessor 0. without church or charity as predecessor	Hospitals’ official websites
*var*7: university affiliation	1. direct affiliation 0. non-direct affiliation	Hospitals’ official websites
*var*8: NHFPC direct administration	1. direct affiliation 0. none direct affiliation	NHFPC website
*var*9: provincial HFPC direct administration	1. direct affiliation 0. none direct affiliation	Websites of the Health and Family Planning Commission of each province
*var*10: municipality HFPC direct administration	1. direct administration 0. none direct administration	http://wjw.beijing.gov.cn/ http://wsjk.tj.gov.cn/html/wsjn/portal/index/index.htm http://wsjkw.sh.gov.cn/index.html http://wsjkw.cq.gov.cn/
*var*11: prefecture bureau of Health and Family Planning Commission administration	1. direct administration 0. none direct administration	Authors’ summary
*var*12: military affiliation	1. military affiliation 0. non-military affiliation	https://www.yaofangwang.com/yiyuan/
*var*13: city administrative level	municipality provincial capital sub-provincial city prefecture	China City Statistical Yearbook 2017

**Table 2:** The Regression Model based on Equation (1).

Linear regression Number of obs = 368 F(21, 346) = 29.16 Prob > F = 0.0000 R-squared = 0.5959 Root MSE = 115.83 Robust					
y	β Coefficients	Standard Error	t	P > t	(95% Confidence Interval)
*var*1	0.0436	0.0080	5.50	0.000 ***	0.0280–0.0592
*var*2	0.0356	0.0203	1.75	0.081 *	−0.0044–0.0755
*var*3	0.0042	0.0008	5.39	0.000 ***	0.0027–0.0058
*var*4 = 1	105.3848	32.6609	3.23	0.001 ***	41.1459–169.6237
*var*5 =					
2	−105.664	18.3378	−5.76	0.000 ***	−141.7317–−69.5964
3	−142.515	40.0669	−3.56	0.000 ***	−221.3204–−63.7096
*var*6 = 1	44.8862	16.5621	2.71	0.007 ***	12.3111–77.4613
*var*12 = 1	171.0338	59.9637	2.85	0.005 ***	53.0946–288.9731
*var*9 = 1	23.8974	32.1457	0.74	0.458	−39.3281–87.1230
*var*10 = 1	13.3541	38.2681	0.35	0.727	−61.9132–88.6215
*var*8 * *var*7					
0 1	−94.1936				
1 0	60.0584	34.6160	−2.72	0.007 ***	−162.278–−26.1093
1 1	0 (omitted)	38.9043	1.54	0.124	−16.4603–136.577
*var*11 * *var*7					
0 1	−67.3168				
1 0	159.3906	32.0474	−2.10	0.036 *	−130.3491–−4.2845
1 1	0 (omitted)	39.2444	4.06	0.000 ***	82.2029–236.5783
*var*13 * *var*7					
1 1	257.8345	64.8942	3.97	0.000 ***	130.1978–385.4712
2 0	59.5823	59.4744	1.00	0.317	−57.3946–176.5593
2 1	283.7637	72.0074	3.94	0.000 ***	142.1364–425.391
3 0	−24.1865	66.7344	−0.36	0.717	−155.4427–107.0697
3 1	300.5646	96.5215	3.11	0.002 ***	110.7219–490.4072
4 0	−25.5099	65.3923	−0.39	0.697	−154.1263–103.1066
4 1	270.0733	80.1628	3.37	0.001 ***	112.4057–427.741
_cons	63.0924	77.3488	0.82	0.415	−89.0406–215.2255

Note: The subscripts *, **, and *** refer to significance levels for two-tailed tests at *p* < 0.1, *p* < 0.05, and *p* < 0.01, respectively. For dummy and categorical variables, we notice the confidence intervals are somewhat wide in the result, which is mainly caused by the small and imbalanced sample size (especially for categorical variables). This can only be improved with a larger sample size or the inclusion of other key variables once additional relevant data and information becomes available.

**Table 3:** Top 10 Cities Ranked by City’s Aggregated 3-A Hospital Score vs. Average 3-A Hospitals Score Per 10,000 People.

	City’s Aggregated 3-A Hospital Score			Average 3-A Hospitals Score Per 10,000 People		
Ranking	City Name	City Administrative Level	Province	City Name	City Administrative Level	Province
1	Beijing	Municipality	Beijing	Xining	Provincial capital	Qinghai
2	Guangzhou	Provincial capital	Guangdong	Urumqi	Autonomous capital	Xinjiang
3	Shanghai	Municipality	Shanghai	Nanchang	Provincial capital	Jiangxi
4	Nanjing	Provincial capital	Jiangsu	Nanjing	Provincial capital	Jiangsu
5	Wuhan	Provincial capital	Hubei	Taiyuan	Provincial capital	Shanxi
6	Xi’an	Provincial capital	Shaanxi	Shenyang	Provincial capital	Liaoning
7	Hangzhou	Provincial capital	Zhejiang	Xi’an	Provincial capital	Shaanxi
8	Shenyang	Provincial capital	Liaoning	Lhasa	Autonomous capital	Tibet
9	Chengdu	Provincial capital	Sichuan	Beijing	Municipality	Beijing
10	Harbin	Provincial capital	Heilongjiang	Guangzhou	Provincial capital	Guangdong

Source: compiled by the authors.

**Table 4:** Total GE (α) Values Based on City’s Average Score Per 10,000 People (at city level).

Grouping at City Level *N* = Observation, aw: Analytical Weight	Theil’s L GE(0)	Theil’s T GE(1)
City (*N* = 308, aw: city population)	0.337	0.330
Subgroups by city administrative level (*N *= 308, aw: city population)	Theil’s L GE(0)	Theil’s T GE(1)
2-Provincial capital 3-Sub-provincial leveled city 4-Prefecture	0.055 0.091 0.184	0.052 0.098 0.175
GE_W(α) GE_B(α)	47.52% 52.47%	35.93% 64.07%

Source: Calculated by the authors. Note: GE_W(α)denotes the within-group inequality for α=0 or 1, and GE_B(α) denotes the between-groups inequality for α=0 or 1.

**Table 5:** Total GE(α) Values based on City’s average score per 10,000 People (subgroups by province, region and zone).

32 Provinces	GE(0)	GE(1)	7 Regions	GE(0)	GE(1)	3 Zones	GE(0)	GE(1)
Total	0.34	0.32	Total	0.34	0.32	Total	0.34	0.32
1-Heilongjiang	0.28	0.18	1-NE	0.32	0.25	1-Eastern China	0.28	0.27
2-Jilin	0.38	0.32	2-NC	0.39	0.36	2-Central China	0.37	0.35
3-Liaoning	0.30	0.27	3-EC	0.25	0.25	3-Western China	0.37	0.38
4-Beijing	0	0	4-CC	0.38	0.38			
5-Tianjin	0	0	5-SC	0.26	0.26			
6-Hebei	0.18	0.15	6-SW	0.28	0.28			
7-Shanxi	0.43	0.47	7-NW	0.42	0.37			
8-Inner Mongolia	0.34	0.36						
9-Shanghai	0	0						
10-Jiangsu	0.37	0.36						
11-Zhejiang	0.19	0.21						
12-Anhui	0.19	0.18	Note: 1-NE comprises provinces No. 1~3; 2-NC comprises provinces No. 4~8; 3-EC comprises provinces No. 9~14; 4-CC comprises provinces No. 15~18; 5-SC comprises provinces No. 19~21; 6-SW comprises provinces No. 22~26; 7-NW comprises provinces No. 27~32.			Note: 1-Eastern China comprises provinces No. 3, 4, 5, 6, 9, 10, 11, 13, 14, 19, 20 and 21; 2-Central China comprises provinces No. 1, 2, 8, 12, 15, 16, 17 and 18; 3-Western China comprises provinces No. 22, 23, 24, 25, 26, 27, 28, 29, 30, 31 and 32.		
13-Fujian	0.27	0.26						
14-Shandong	0.18	0.18						
15-Henan	0.35	0.30						
16-Hubei	0.34	0.32						
17-Hunan	0.26	0.28						
18-Jiangxi	0.52	0.57						
19-Guangdong	0.24	0.26						
20-Hainan	0.002	0.002						
21-Guangxi	0.27	0.25						
22-Chongqing	0	0						
23-Sichuan	0.28	0.26						
24-Guizhou	0.41	0.46						
25-Yunnan	0.31	0.34						
26-Tibet	0	0						
27-Shaanxi	0.43	0.39						
28-Gansu	0.33	0.34						
29-Qinghai	0	0						
30-Ningxia	0	0						
31-Xinjiang	0.43	0.33						
32-XPCC	0	0						
GE_W(α) GE_B(α)	80.36% 19.64%	76.13% 23.87%	GE_W(α) GE_B(α)	92.82% 7.18%	92.52% 7.47%	GE_W(α) GE_B(α)	97.43% 2.56%	97.35% 2.65%

Source: Calculated by the authors. Note: GE(α) Values are all calculated based on 312 observations, which combines 308 cities and 4 municipalities.
